# Elevated and sustained anti‐feeding effect of Scalibor® deltamethrin collar against the sand fly *Phlebotomus perniciosus* in dogs confirmed for 1 year following treatment

**DOI:** 10.1111/mve.12545

**Published:** 2021-08-27

**Authors:** A. Evans, G. Bongiorno, J. J. Fourie, N. Lekouch, R. Bianchi, C. Khoury, E. Thomas, R. Chiummo, L. Gradoni

**Affiliations:** ^1^ Clinvet SA Morocco Mohammedia Morocco; ^2^ Unit of Vector‐borne Diseases, Department of Infectious Diseases Istituto Superiore di Sanità Rome Italy; ^3^ Clinvet International (Pty) Ltd Bloemfontein South Africa; ^4^ MSD Animal Health Innovation GmbH Schwabenheim Germany

**Keywords:** anti‐feeding efficacy, deltamethrin collar, dog, fast‐killing efficacy, *Phlebotomus perniciosus*

## Abstract

Dogs are reservoir hosts for *Leishmania infantum*, a protozoan parasite transmitted by phlebotomine sand flies. The anti‐feeding and fast‐killing efficacy of Scalibor® deltamethrin collars against experimental *Phlebotomus perniciosus* challenges on dogs was determined over 1 year. Two groups of 8 dogs each were fitted with placebo (control) or deltamethrin collars (treated) on Day 0 and exposed to sand flies approximately every 28 days up to Day 364. After each exposure, anti‐feeding and fast‐killing efficacy rates were determined by comparing blood‐fed or live insects, respectively, in the treated vs. the control group. Blood‐fed and live sand flies were significantly less in treated dogs as compared to control dogs at each assessment. The anti‐feeding efficacy rate exceeded 90% except on Day 337 (89%) but increased again (96%) on Day 364. Fast killing efficacy was <74% over the study when considering all flies. However, this value increased cumulatively to 98% when only blood‐fed flies were compared between groups. Scalibor® collars are highly effective at preventing *P. perniciosus* blood‐feeding and in fast‐killing flies taking a blood meal for up to 1 year after application. These strong and long‐lasting effects are an important strategic component for *L. infantum* transmission control.

## Graphical abstract

The anti‐feeding and fast‐killing efficacy of Scalibor® deltamethrin collars against experimental *Phlebotomus perniciosus* challenges on dogs was determined over 1 year.
After each exposure, anti‐feeding and fast‐killing efficacy rates were determined by comparing means of blood‐fed or live sand flies, respectively, in the treated vs. the control group (eight dogs each).
Anti‐feeding efficacy exceeded 90% in 12/13 determinations performed over 364 days, whereas fast killing efficacy reached 98% when only blood‐fed sand flies were compared between treated and control dogs.

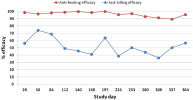

## Introduction

Canine leishmaniosis (CanL) is a protozoan infection due to *Leishmania infantum* (synonym: *L. chagasi*) (Kinetoplastida: Trypanosomatidae) endemic in the Mediterranean littoral, Middle East, Central Asia and Latin America (Moreno & Alvar, 2002; Dantas‐Torres *et al*., 2012). The infection may result in apparently healthy condition for years or in overt disease with different degrees of severity, from mild to fatal pathological signs. Early in the chronic progression of CanL, lymphadenopathy and weight loss are the most common signs, followed by skin (exfoliative/ulcerative dermatitis, alopecia, onychogryphosis), ocular signs and life‐threatening renal alterations (Foglia Manzillo *et al*., 2013). Although the parasite can occasionally be transmitted dog‐to‐dog through sexual, congenital or blood‐transfusion routes (reviewed in EFSA Panel on Animal Health and Welfare, 2015), the natural mode is through skin inoculation of *Leishmania* infective stages (metacyclic promastigotes) by infecting phlebotomine vectors at the time of taking a blood meal on the canine host (Bates, 2007).

*Phlebotomus perniciosus* (Diptera: Psychodidae) is a proven vector of *L. infantum* in the western and central Mediterranean subregion, including Portugal, Spain, southern France, Italy, and northwest Croatia in the EU, and Morocco, Algeria, and Tunisia in north Africa (European Centre for Disease Prevention and Control, 2020). Recently, this sand fly species was shown to have potential competence in the transmission of *Leishmania tropica* (Bongiorno *et al*., 2019), an occasional agent of CanL in north Africa and the Middle East (Baneth *et al*., 2017). *P. perniciosus* is a major member of the *Phlebotomus* (*Larroussius*) subgenus, which includes close‐related sand fly species involved in CanL transmission in parts of the Mediterranean littoral, namely *Phlebotomus ariasi*, *Phlebotomus neglectus* and *Phlebotomus tobbi* (Alten *et al*., 2016).

Dog protection from sand fly bites is the optimal first‐line approach to prevent CanL infections and transmission of zoonotic visceral leishmaniasis by infected dogs (World Health Organization, 2010). This can be achieved with several topical medications, such as collars, spot‐ons or sprays, containing synthetic pyrethroids proven to have anti‐feeding (excito‐repellent) and insecticidal effects against phlebotomine sand flies. Deltamethrin‐impregnated collars were the first canine topical formulation that showed elevated and prolonged anti‐feeding and insecticidal effects against *P. perniciosus*, seen initially in a 34‐week study which included two treated and two control dogs (Killick‐Kendrick *et al*., 1997). Thereafter, other laboratory studies confirmed the efficacy of deltamethrin‐impregnated collars against phlebotomine vectors of *L. infantum* in the New World, *Luztomyia longipalpis*, and *Luztomyia* (syn. *Migonemyia*) *migonei* (David *et al*., 2001). Recently, a laboratory study challenging 16 dogs with *P. perniciosus*, proved that the application of deltamethrin collars conferred ≥ 94% protection from sand fly blood‐feeding when compared to untreated animals over the full year (Paulin *et al*., 2018). The study used batches of laboratory‐reared sand flies obtained from the Department of Parasitology of Charles University in Prague, Czech Republic. The objective of the present study was to confirm this result using a different origin sand fly, *P. perniciosus* specimens reared at Istituto Superiore di Sanità in Rome, Italy.

## Materials and methods

This was a parallel‐group, single‐centre, placebo‐controlled, randomized, and masked efficacy study, designed to determine the anti‐feeding effect, as primary efficacy criterion, of deltamethrin collars (Scalibor® protector band, MSD Animal Health) against experimental *P. perniciosus* challenges on Beagle dogs. The fast‐killing effect was also evaluated as a secondary efficacy criterion; ‘fast‐killing’ rather than ‘insecticidal’, because the latter evaluation requires 24 h after product exposure of sand flies (Alexander & Maroli, 2003). In this study, the sand flies were examined immediately after the challenge in order to accurately determine the blood‐feeding status through the immobilization of live flies (see below). This study was reviewed and approved by the Institutional Animal Care and Use Committee of Clinvet and Ethics Committee of MSD Animal Health Innovation and conducted following the international guidelines on animal welfare.

### Sand flies

Adult laboratory‐bred *P. perniciosus* specimens, females and males from a Spanish (Madrid, MD, U.S.A.) colony strain, were maintained at the insectarium of Istituto Superiore di Sanità since June 2012. Periodical PCR analysis of random flies for *Leishmania* and *Phlebovirus* confirmed the strain as a Specific Pathogen Free. Sand flies were shipped by air to Clinvet laboratories (Morocco) within pots containing the required number of specimens for each dog, as recently described (Bongiorno *et al*., 2020). At the time of the challenge, sand flies' age was in the range of 7–14 days.

### Dog inclusion, treatment and husbandry

Twenty male and female Beagles aged >6 months at enrolment were acclimatized before the study for a maximum of 22 days, during which period all care procedures and conditions were standardized and remained similar throughout the study. Dogs did not receive topical or systemic administration of insecticidal products during 12 weeks preceding the treatment day (Day 0). During acclimatization, 31 dogs were challenged with female *P. perniciosus* (from 40 to 75 specimens) to ensure selected dogs presented an adequate attractiveness to sand flies. Sixteen dogs from this group were included in the study and allocated to treat and placebo‐controlled groups based on total counts of fed sand flies and by dog sex. All dogs were fitted with plastic non‐medicated collars prior to Day 0 to acclimate to this procedure. On treatment Day 0, the non‐medicated plastic collars were removed, and eight dogs received deltamethrin‐impregnated collars (Scalibor® protector band) at the label recommended dose following application instructions, and a placebo collar was applied to the remaining eight dogs (control group). Enrolled dogs were housed separately in a semi‐indoor animal unit and exposed to ambient temperatures and natural light conditions throughout the study. The dogs received an age‐appropriate standard commercial dog diet and potable water *ad libitum* and were allowed controlled and monitored access to an outdoor, shared exercise area for a minimum of 15 min per day.

### Sand fly challenge, efficacy assessments and statistics

Prior to the sand fly challenge, sand flies were released into challenge cages and allowed to acclimatize for approximately 30 min in the dark. The cages were made of fine mesh netting mounted on a metal frame of appropriate size (81 cm length, 58 cm width and 58 cm height), which is standard for studies on canine topical pyrethroids using sand flies (Molina *et al*., 2012) (cage size: 75 cm × 50 cm × 50 cm) or mosquitoes (Franc *et al*., 2012) (cage size: 60 cm × 40 cm × 50 cm). Dogs were sedated with medetomidine hydrochloride and subsequently transferred to the challenge room, where they were placed individually into challenge cages. The whole body of the dog was exposed to unfed sand flies. Challenge started when lights were turned off and ended when lights were turned on. Each dog was exposed to approximately 85 female and 10 male flies for 60 min (±5) at intervals of approximately 28 days: Days 28, 56, 84, 112, 140, 168, 197, 224, 253, 280, 308, 337 and 364. Room temperatures were maintained at ∼25 °C, and relative humidity, 60 to 80%, were recorded throughout the exposure.

At the end of each exposure, all live (able to fly normally) sand flies were collected from challenge cages into a vented container using a battery‐powered mechanical aspirator. Each dog was checked before and after removal from the cage to collect any visible flies, either live, moribund (uncoordinated movements and unable to fly) or dead. Compressed air was blown into the dog's hair to aid recovery of remaining insects, and dead sand flies were placed in a separate container. CO_2_ or freezing was used to permit engorgement status evaluation of live females. Females were categorized as fed or unfed visually or, if blood engorgement status was unclear, the gut was microscopically inspected for blood traces. Anti‐feeding and fast‐killing efficacy values (%) were calculated using the arithmetic means of blood‐engorged or live/moribund females, respectively, according to the following formula:

%Efficacy=100×[(Mc−Mt)/Mc]

where Mc is the arithmetic mean count of the control group, and Mt is the arithmetic mean count of the treated group.

Two‐tailed statistical tests at 5% level of significance were performed using the software package SAS® (SAS Institute Inc., Cary, NC, U.S.A.). Group sand fly counts were analysed by one‐way anova with a treatment effect on both untransformed and logarithmically transformed data, and the non‐parametric Mann‐Whitney test.

## Results

On Day 21, one dog in the deltamethrin‐treated group lost and damaged its collar, and this dog was excluded from subsequent challenges and assessments. Another dog from the same group destroyed its collar on Day 355 and was excluded from Day 364 assessment. Dermatitis of the neck was reported in two treated group dogs in the first 2 months of collar application. Both dogs recovered with symptomatic treatment.

**Fig. 1:**
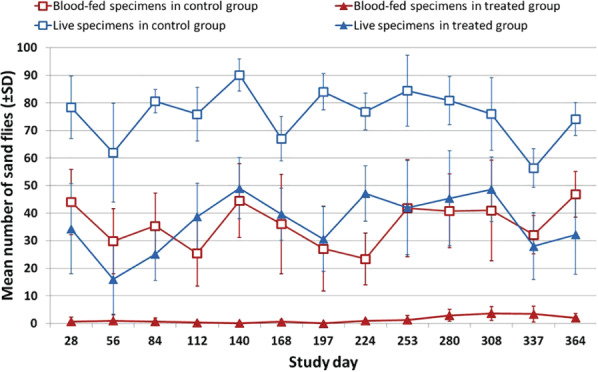
Blood‐fed and live sand flies (*Phlebotomus perniciosus*) after exposure to deltamethrin‐treated or control dogs throughout the one‐year challenge period.

The number of blood‐fed sand flies and the number of live sand flies in the treated group was significantly lower when compared to the placebo control group after all challenges (*P* < 0.01 as calculated for both one‐way anova and Mann‐Whitney test) (Tables 1 and 2; Fig. 1).

**Table 1:** Blood‐engorged sand fly counts after challenge of control (placebo collar) or treated (deltamethrin collar) dogs.

	Mean no. of engorged sand flies (range)			
Challenge day	Placebo	Deltamethrin	*P* value (one‐way anova/Mann‐Whitney test)	% anti‐feeding efficacy
28	44.0 (25–62)	0.7 (0–4)	<0.0001/0.0012	98.4
56	29.8 (14–46)	1.0 (0–6)	<0.0001/<0.0012	96.6
84	35.4 (22–56)	0.7 (0–3)	<0.0001/<0.0012	98.0
112	25.4 (5–37)	0.3 (0–1)	<0.0001/<0.0012	98.9
140	44.5 (25–67)	0.1 (0–1)	<0.0001/<0.0010	99.7
168	36.0 (17–63)	0.6 (0–2)	0.0002/0.0013	98.4
197	27.1 (7–48)	0.0 (0–0)	0.0005/0.0008	100.0
224	23.4 (12–35)	1.0 (1–1)	<0.0001/0.0008	95.7
253	41.8 (24–73)	1.3 (0–4)	<0.0001/0.0014	96.9
280	40.8 (23–62)	2.9 (0–5)	<0.0001/0.0014	93.0
308	41.0 (20–68)	3.6 (0–7)	0.0001/0.0014	91.3
337	32.1 (17–40)	3.4 (0–9)	<0.0001/0.0014	89.3
364	46.8 (39–66)	2.0 (0–4)	<0.0001/0.0023	95.7

**Table 2:** Live (including moribund) sand fly counts after challenge of control (placebo collar) or treated (deltamethrin collar) dogs.

	Mean no. of live sand flies (range)			
Challenge day	Placebo	Deltamethrin	*P* value (one‐way anova/Mann‐Whitney test)	% fast‐killing efficacy
28	78.4 (60–93)	34.3 (18–57)	<0.0001/0.0014	56.3
56	61.9 (29–76)	16.0 (4–36)	<0.0001/0.0032/	74.1
84	80.6 (76–86)	25.1 (17–44)	<0.0001/0.0014	68.8
112	75.9 (61–95)	38.7 (27–61)	<0.0001/0.0017	49.0
140	90.1 (81–100)	49.0 (33–65)	<0.0001/0.0015	45.6
168	67.0 (57–76)	39.6 (27–57)	<0.0001/0.0017	40.9
197	84.0 (74–95)	30.6 (11–41)	<0.0001/0.0015	63.6
224	76.8 (66–83)	47.1 (33–58)	<0.0001/0.0014	38.6
253	84.4 (61–103)	42.0 (22–72)	0.0001/0.0022	50.2
280	80.9 (66–93)	45.4 (25–69)	0.0002/0.0021	43.8
308	76.0 (51–94)	48.6 (32–63)	0.0010/0.0065	36.1
337	56.3 (48–68)	28.0 (14–43)	<0.0001/0.0014	50.2
364	74.1 (65–81)	32.2 (14–55)	<0.0001/0.0024	56.6

**Fig. 2:**
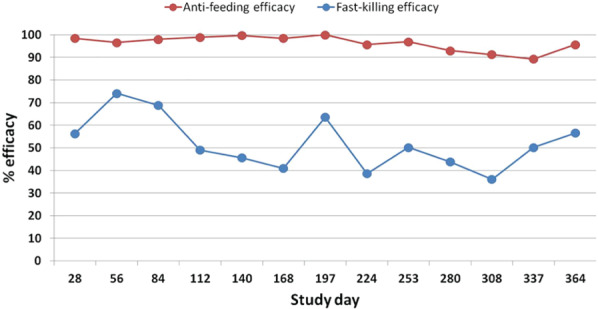
Anti‐feeding and fast‐killing efficacies (%) against sand flies (*Phlebotomus perniciosus*) calculated from comparative results of deltamethrin‐ or placebo‐treated dogs.

Anti‐feeding efficacy was >90% at all time points except on Day 337 (89.3%.) and reached 100% on at least one assessment. At the last time point on Day 364, the anti‐feeding efficacy was >95% (Table 1; Fig. 2). Fast‐killing efficacy values were ≤74% over the challenge period, comparing blood‐fed and unfed flies (Fig. 2). The deltamethrin collar exhibited much higher fast‐killing efficacy when only blood‐fed sand flies were compared between treated and control dogs. The total number of live blood‐fed specimens was 3622 in the placebo group and 72 in the treated group, and the cumulative fast‐killing efficacy was calculated to be 98.0% over the study period (a range of 93.3–100%).

## Discussion

Results of this study confirm, using a different sand fly (*P. perniciosus)* population, the previous assessment (Paulin *et al*., 2018) that Scalibor® collars deliver a potent and rather constant anti‐feeding activity over 12 months. A slightly broader anti‐feeding efficacy rate was observed during this study (89.3–100%) compared with the previous one (94–98%). On the other hand, fast‐killing activity recorded throughout the year markedly differed between the two studies: the earlier study observed a decreased fast‐killing efficacy from the fourth month of treatment, becoming negligible from the sixth month onwards, while the present study reports variable and higher efficacy (36.1–74.1%) until the study end reaching 56.6% on Day 364 (Table 2 and Fig. 1). This difference may indicate a variation in deltamethrin susceptibility between the two *P. perniciosus* in the studies or, possibly, to variations in the methods used to recover dead sand flies after the challenge. Findings from the present study agree with those from the first published study on Scalibor® collars and sand flies (Killick‐Kendrick *et al*., 1997), where sustained *P. perniciosus* lethality was observed at 2 h exposure in assessments performed for up to 34 weeks (about 8 months) on two treated dogs. The 1997 study observed variable mortality rates (25–64%) over the whole study period, with a rate of 55–60% recorded on the last assessment (week 34), resulting in 71% fast‐killing efficacy – unreported in the paper and calculated from published raw data including fly mortality values from two untreated control dogs.

In our study, we observed a very strong fast‐killing effect (98%) against sand flies taking a blood meal from treated dogs. This effect, thought to result from longer exposure of biting female flies to the treated dog, was not analysed in the results of Paulin *et al*. (2018). It may have an important role in blocking further transmission from any *Leishmania*‐infected dogs treated with a deltamethrin collar. The combined effect of individual protection from potential infective bites and control of further transmission rate from infected dogs can explain the efficacy of deltamethrin collars to control this zoonosis. An international panel of experts (Dantas‐Torres *et al*., 2020) reviewed the available scientific data supporting the treatment of dogs with insecticide‐impregnated collars to decrease the risk of *L. infantum* infection transmission among dogs and people living in areas endemic for visceral leishmaniosis. Many prior papers share a similar conclusion (Maroli *et al*., 2010; Miró *et al*., 2017; Dantas‐Torres *et al*., 2019, among others), including a scientific opinion by the European Food Safety Authority (EFSA Panel on Animal Health and Welfare, 2015). Several field studies, using various methodologies, found that deltamethrin collar provides a median 53.5% protection (range: 42.4–100%) against canine *Leishmania* seroconversion in dog populations across endemic regions of Old and New Worlds (reviewed by Dantas‐Torres *et al*., 2020). A recent meta‐analysis that systematically reviewed the relatively limited literature also reached a similar conclusion, estimating an overall 54% efficacy in decreasing CanL incidence worldwide (Yimam & Mohebali, 2020). Furthermore, widespread application of deltamethrin collars to dogs provided a 50% reduction (95% CL: 30%, 82%) of infantile visceral leishmaniasis in a randomized controlled trial including 80 villages in a Mediterranean biotope of northwest Iran (Courtenay *et al*., 2019).

Pyrethroid‐impregnated collars provide a slow release of the active ingredient and are optimally suited for long‐term control programmes against zoonotic visceral leishmaniosis. A comparative efficacy study of flumethrin (Seresto®, Elanco Animal Health, Indianapolis, IN, U.S.A.) and deltamethrin collars against CanL over one transmission season in dogs from a Mediterranean area of high endemicity showed that both products were significantly effective in preventing *L. infantum* infection (Brianti *et al*., 2016). Whereas laboratory data on the efficacy duration of flumethrin collars are not available in scientific literature, results of the study reported here indicate that a single deltamethrin collar can contribute to the sand fly control that is needed to reduce *L. infantum* transmission for 1 year. The activity period of adult females of *Phlebotomus* (*Larroussius*) spp. in southern Mediterranean settings of CanL was recently found to be as long as 8–9 months, i.e. from April through November/December (Lisi *et al*., 2014; Alten *et al*., 2016) on the other hand 5–6 months, i.e. from May through September/October, recorded in the early 1990s (Killick‐Kendrick *et al*., 1997).

## Conclusions

Deltamethrin releasing collar worn continuously by the dogs for up to 1 year provides highly effective anti‐feeding activity against sand flies (*P*. *perniciosus*) challenge. There is also a measurable fast‐killing effect that is more apparent in blood‐fed flies over the one‐year treatment period.

## Author contributions

AE, JJF, ET, RC and LG designed the study; GB, RB and CK performed mass sand fly rearing; AE, GB, NL and LG performed the experiments on dogs; AE and LG drafted the manuscript; all authors reviewed the manuscript.

### Data availability statement

The data that support the findings of this study are available from the corresponding author upon reasonable request.
